# The efficacy of sodium benzoate as an adjunctive treatment in early psychosis - CADENCE-BZ: study protocol for a randomized controlled trial

**DOI:** 10.1186/s13063-017-1908-5

**Published:** 2017-04-07

**Authors:** Alex Ryan, Andrea Baker, Frances Dark, Sharon Foley, Anne Gordon, Sean Hatherill, Stephen Stathis, Sukanta Saha, George Bruxner, Martin Beckman, Drew Richardson, Michael Berk, Olivia Dean, John McGrath, Cadence Working Group, James Scott

**Affiliations:** 1 Queensland Centre for Mental Health Research, The Park Centre for Mental Health, Wacol, QLD Australia; 2 University of Queensland Centre for Clinical Research, University of Queensland, Herston, QLD Australia; 3 Metro South Mental Health, 519 Kessels Road, MacGregor, QLD 4109 Australia; 4 Metro North Mental Health, Royal Brisbane and Women’s Hospital, Level 3 UQCCR, RBWH, Herston, QLD 4029 Australia; 5 Logan Hospital, Armstrong Rd and Loganlea Rd, Meadowbrook, QLD 4131 Australia; 6 Lady Cilento Children’s Hospital, Raymond Terrace, South Brisbane, QLD 4101 Australia; 7 Queensland Brain Institute, University of Queensland, St Lucia, QLD Australia; 8 Metro North Mental Health, Caboolture and Redcliffe Hospitals, Caboolture, QLD Australia; 9 Specialist Child and Adolescent Psychiatrist, Evolve Therapeutic Services Logan, Child and Youth Mental Health Services Logan, Academic Clinical Unit Logan, Metro South Hospital and Health Services, Logan, QLD Australia; 10 Prince Charles Hospital, Chermside, Rode Rd, Chermside, QLD 4032 Australia; 11 IMPACT Strategic Research Centre, Deakin University, School of Medicine, Barwon Health, P.O. Box 291, Geelong, 3220 Australia; 12 Department of Psychiatry, University of Melbourne, Royal Melbourne Hospital, Level 1 North, Main Block, Parkville, 3052 Australia

**Keywords:** Adjuvant, Sodium benzoate, Early Psychosis, Schizophrenia, Intervention, RCT, Clinical trial, PANSS

## Abstract

**Background:**

Psychotic disorders affect up to 3% of the population and are often chronic and disabling. Innovation in the pharmacological treatment of psychosis has remained stagnant in recent decades. In order to improve outcomes for those with psychotic disorders, we present a protocol for the trial of a common food preservative, sodium benzoate, as an adjunctive treatment in early psychosis.

**Methods:**

Persons experiencing early psychosis (n = 160) will be recruited through hospitals and community mental health services in Queensland, Australia. Patients will be randomized to receive either 12-week treatment with 1000 mg (500 mg twice daily (BD)) sodium benzoate or placebo. Patients will undergo fortnightly outcome assessments, in addition to weekly ongoing capacity to consent, drug compliance and safety assessments. The primary outcome measure is the Positive and Negative Syndrome Scale (PANSS) total score. Secondary outcomes are Global Assessment of Function (GAF), Assessment of Quality of Life Scale (AQOL), the Activity and Participation Questionnaire (APQ6), International Physical Activity Questionnaires (IPAQ), Simple Physical Activity Questionnaire (SIMPAQ), Physical Activity Questionnaire, Clinical Global Impression (CGI), Hamilton Depression rating Scale-17 items (HDRS), Opiate Treatment Index (OTI) and the Patients’ Global Impression of Improvement (PGI-I). As a tertiary objective, changes from baseline to endpoint in to serum markers related to D-alanine, L-alanine, D-serine, L-serine, glycine and glutamate will be investigated.

**Discussion:**

Consumers and clinicians are keen to help develop better treatments for those with psychosis. This study, part of the wider Cadence clinical trials platform will examine if a safe and accessible food preservative can help optimize outcomes in those with psychosis.

**Trial Registration:**

Australian New Zealand Clinical Trials registry (ANZCTR), ACTRN12615000187549. Registered on 26 February 2015.

**Electronic supplementary material:**

The online version of this article (doi:10.1186/s13063-017-1908-5) contains supplementary material, which is available to authorized users.

## Background

Psychotic disorders are characterized by disturbances to cognition, affect, perception and behaviour. Approximately three percent of people in the population are affected by psychosis; onset is commonly in the second or third decade of life with male individuals at higher risk. While antipsychotic drugs and supportive therapies reduce the symptom burden and rate of relapse to a certain extent, many will have persistent symptoms and concomitant cognitive and social disabilities, poor physical health and curtailed life expectancy [1, 2]. The pharmacological treatment of psychotic disorders has seen little innovation in recent decades. No treatment is documented as efficacious for primary negative symptoms and cognitive impairments. In addition, current medications for psychosis are associated with side effects such as weight gain and metabolic syndrome. Pharmacological therapy for psychosis yields inconsistent results with a sizeable proportion of patients only partially responding to treatment or remaining treatment-resistant [3]. To reduce the personal, social and economic burden associated with psychotic disorders, it is imperative to investigate novel methods of therapeutic treatment in psychosis.

## Novel interventions for psychosis

Traditionally pharmacological treatments of psychotic illness have predominately focused on modulating the dopaminergic system. However, recent evidence suggests that modulation of the glutamatergic system also plays a vital role as has been shown by abnormalities in glutamatergic neurotransmission mediated by N-methyl-D-aspartate (NMDA) (a subtype of glutamate receptors) in schizophrenia [4]. NMDA receptors can be simplified into two main subunits, the glutamate and glycine binding sites. Options for enhancing NMDA function are limited to agonists or modulators of the glycine binding site, as increasing glutamate levels causes excitotoxicity of neural cells.

The D-amino-acids, D-serine and D-alanine, act as full agonists of the glycine binding site and have shown some promise as an adjunct therapy for treatment of schizophrenia [5]. On meta-analysis [5], the use of D-serine as an adjunct with antipsychotics was found to improve total psychopathology (Cohen’s *d* effect size and 95% confidence intervals) 0.40 (0.07–0.73), negative symptoms, 0.48 (0.06–0.90) and notably, also to improve cognitive symptoms 0.42 (0.12–0.73). D-serine also produced a borderline significant improvement in depressive symptoms 0.39 (−0.01–0.79); however, it had no effect on positive symptoms or general psychopathology. D-alanine has only been investigated in one randomized controlled trial as an adjunct therapy in patients with schizophrenia and has shown similar promise to that of D-serine [6].

A limiting factor with the use of D-serine and D-alanine as adjunct treatments are their oxidization by a flavoenzyme, D-amino acid oxidase (DAAO) [7, 8]. This process both limits the bioavailability of these amino acids, lessening their effectiveness, and increases products that are potentially nephrotoxic in high doses [9, 10]. This limitation has led to interest in compounds that may inhibit the activity of DAAO, and thus lead to an accumulation of endogenous D-alanine. One inhibitor of DAAO is the widely used food preservative sodium benzoate. Experimental animal studies have confirmed that sodium benzoate diminishes DAAO’s ability to oxidize D-serine and D-alanine, resulting in increased cerebral concentration of these D-amino acids [11].

## Rationale for the use of sodium benzoate for the treatment of schizophrenia

In a recent clinical trial, 52 patients with chronic schizophrenia were assigned to adjunct placebo or adjunct sodium benzoate (1000 mg per day) treatment for a period of 6 weeks [12]. At the end of the study period all domains of the Positive and Negative Syndrome Scale (PANSS) had improved by an average of 21% (with effect sizes (ES) of 1.16–1.69) and neurocognitive testing showed improved processing speed (*P* = 0.03, ES = 0.65) and visual learning and memory (*P* = 0.02, ES = 0.70). Other domains remained unchanged. There was no increase in adverse effects in the sodium benzoate or the control group. In the benzoate group there was one recorded case of tachycardia, one of weight gain and two of insomnia. The authors suggested these events were likely coincidental observations [12].

There is also evidence related to the use of sodium benzoate in other mental disorders. A randomized, double-blind, placebo-controlled trial investigating the use of sodium benzoate in patients with Alzheimer disease was identified [13]: 60 patients with amnestic mild cognitive impairment or mild Alzheimer disease were treated with a variable dose of sodium benzoate (250–750 mg/day) for a period of 24 weeks. Patients treated with sodium benzoate performed better in the cognitive subscale of the Alzheimer’s disease Assessment Scale (*P* = 0.0031) than those on placebo, and in the cognition composite (*P* = 0.0007) and the clinician interview-based impression of change plus caregiver input (*P* = 0.012) [13]. It was also noted that sodium benzoate was well-tolerated without any evident side effects.

Sodium benzoate has also been reported in three case studies. Each patient was administered a 6-week trial of sodium benzoate (500 mg per day) in patients who had refused conventional medications. Two of these were drug-naïve patients with major depression. Over the 6-week course there was a reduction in the Hamilton Rating Scale for Depression (Ham-D) score in the first patient, which fell from 25 to 9; likewise, in the second patient with depression, the depression scores dropped after treatment [14, 15]. Last, a patient experiencing panic disorder with somatic symptoms had a reduction in the Panic Disorder Severity Scale from 18 to 7 and an associated reduction in somatic symptoms [16]. Although these cases are encouraging it is difficult to differentiate improvements due to sodium benzoate from a placebo effect.

In recent decades there has been considerable attention to optimizing treatments during the first few years of onset [17, 18]. Often described as “early psychosis”, this field of research believes that assertive treatments initiated soon after onset may have better outcomes, compared to the same treatments when given to individuals with longstanding, chronic psychotic disorders. To date, we are not aware of studies that have examined the efficacy of sodium benzoate as a treatment in those with early psychosis. In our study, we will include patients within two years of the onset of psychotic disorders.

## Safety profile of sodium benzoate

The acute toxicity of oral sodium benzoate in humans is low. There is evidence that some atopic individuals may be sensitive to food additives and preservatives (benzoate is a food preservative). Thus, participants were screened and excluded when a past history of allergies or intolerance of any food additives was identified (see “Inclusion and exclusion criteria”).

While the use of sodium benzoate for treatment in psychiatric disorders in humans is a contemporary procedure, it has also been used since the late 1970s to treat patients with urea cycle enzymopathies that cause hyperammonaemia [19–21]. The therapeutic dose administered to treat hyperammonaemia over several years is in the range of 250–500 mg/kg body weight per day, which equates to 17,500–35,000 mg per day for a body weight of 70 kg. It is noted that at this dose level, the clinical signs of toxicity are rare and are limited to anorexia and vomiting, especially after large intravenous bolus injections with 100% bioavailability. Recently, Lane et al. [12] used a dose of 1000 mg of sodium benzoate per day in their study with no significant differences in side effects between the treatment and placebo groups.

There is substantial safety information available for sodium benzoate because it is used as a common food preservative for products such as salad dressings, carbonated drinks, jams, fruit juices and other condiments. On labels, the inclusion of sodium benzoate is indicated by the code E211. The US Food and Drug Agency (FDA) has classified sodium benzoate as “Generally Recognized As Safe” and regulates the concentration of sodium benzoate to 0.1% by weight in food products and 1% concentration in medicines [22, 23]. The current acceptable daily intake of 0.5 mg/kg body weight is suggested by the joint committee of the Food and Agriculture Organization of the United Nations (FAO) and the World Health Organization (WHO) [24]. It is noted that intake estimations from several counties are averaged at 0.18–2.3 mg/kg body weight; however, individuals in China can consume up to 14 mg/kg body weight per day from diet alone (i.e. 980 mg per day in a person weighing 70 kg) [25].

The International Programme on Chemical Safety (IPCS) published a report on sodium benzoate (and a related compound, benzoic acid) in 2000 detailing the potential health effects of sodium benzoate in animal studies. Testing in rodents revealed a low rate of toxicity with mean lethal dose (LD50) values >1940 mg/kg body weight. Drawing evidence from two long-term studies (12–16 months) in rodents, there was no evidence to suggest sodium benzoate had carcinogenic properties. Likewise, studies of the precursors of benzoic acid - benzyl acetate, benzyl alcohol, and benzaldehyde - suggest that sodium benzoate is unlikely to have a carcinogenic effect. The results of genotoxic activity were inconclusive in the IPCS report, and there was no consistent abnormal findings based on the Ames test. Based on in vitro studies of human lymphoblastoid cell lines, the evidence suggests that sodium benzoate at very high concentrations does have genotoxic effects.

Sodium benzoate does have embryotoxic and fetotoxic effects; however, these are only evident at a dosage high enough to cause severe maternal toxicity. A no observable adverse effect level (NOAEL) of approximately 1310 mg/kg body weight for teratogenic effects in rodents was established [26].

## Study objectives and hypothesis

The primary objective is to determine if 12-week treatment with 1000 mg (500 mg twice daily (BD)) sodium benzoate improves the PANSS total score compared to placebo. The secondary objectives are to determine if 12-week treatment of 1000 mg (500 mg BD) sodium benzoate improves scores in the PANSS subscales, the Global Assessment of Function (GAF), the Assessment of Quality of Life Scale (AQOL), the Activity and Participation Questionnaire (APQ-6), the International Physical Activity Questionnaires (IPAQ), the Simple Physical Activity Questionnaire (SIMPAQ), the Clinical Global Impression (CGI), the Hamilton Depression rating Scale-17 items (HDRS), the Opiate Treatment Index (OTI) and the Patients’ Global Impression of Improvement (PGI-I), compared to placebo. As a tertiary objective, changes to sera markers related to D-alanine, L-alanine, D-serine, L-serine, glycine and glutamate change from baseline to endpoint will be investigated.

It is hypothesised that 1) participants allocated to the active arm (1000 mg (500 mg BD)) Sodium Benzoate treatment will have significant reductions in PANSS total score at week 12 compared to individuals taking placebo; 2) sodium benzoate treatment will improve scores on secondary scales relative to placebo; and 3) for patients who have received stable primary medication during the trial, only those allocated to the active arm will have modified levels of sera markers related to D-alanine, L-alanine, D-serine, L-serine, glycine and glutamate.

## Study design

The design is a randomized, placebo-controlled, double-blind parallel-group trial to examine the clinical efficacy and safety of add-on treatment with sodium benzoate for persistent symptoms in patients with early psychosis. Standard Protocol Items: Recommendations for Interventional Trials (SPIRIT) guidelines and checklists were followed for this protocol [27, 28] (Additional file 1: Table S1).

In this study 160 individuals with early psychosis will be recruited. Participants will be given either 1 g/d (500 mg BD) of sodium benzoate or placebo (microcrystalline cellulose in matched gelatine capsules), in addition to their normal routine care. Routine care is defined as “individualized combinations of psychopharmacology, behavioural interventions, rehabilitation and associated clinical services” in keeping with Queensland Health standards of care. Face-to-face clinical assessments will be at baseline (week 0) and weeks 2, 4, 6, 8, 10 and 12. Weekly phone contact (if no phone then face-to-face assessment) will occur in between face-to-face visits. A post-completion visit will be conducted at week 14. Participants will be contacted regularly by weekly telephone calls to monitor adherence and compliance.

## Allocation sequence and concealment

Randomization will be carried out using a computer-generated randomization table, stratified by five sites. Each of the five sites will have separate randomization tables, and each list will ensure randomization is blocked in groups of four. Participants will receive either active treatment or placebo in a 1:1 ratio. The allocation sequence will be generated by an independent statistician, and the distribution of the study medication will be supervised by an independent research pharmacist.

## Study population

One hundred and sixty participants will be recruited through hospitals and community mental health services in Queensland, Australia.

## Inclusion and exclusion criteria

The inclusion criteria include: (1) onset of a psychotic disorder within the last 2 years; (2) diagnosis of psychotic disorders according to the Diagnostic and Statistical Manual of Mental Disorders, 4th. Edition (DSM-IV), which include schizophrenia, schizophreniform psychosis, delusional disorder, bipolar disorder and psychosis not otherwise specified; (3) age between 15 and 45 years; 4) treatment with antipsychotic medications for a period of at least one continuous month within the 2-year period as in “(1)”; (5) PANSS total score of at least 55 (i.e. those who have at least moderate symptoms) [29, 30] and (6) agreement to participate, and capacity to consent and be able to follow the study instructions and procedures.

The exclusion criteria include: (1) known allergies to sodium benzoate (E211) or any part of the formulation of the investigational product; (2) suspected allergies or known adverse reactions to food preservatives in general; (3) comorbid physical illnesses that would impair the participant’s ability to complete the trial (e.g. a general medical disorder requiring additional treatments and/or hospitalization); (4) inability to understand or communicate in English; (5) currently pregnant, planning to become pregnant or lactating during the study period and (6) inability to follow the study instructions and procedures.

## Sample size determination

The study by Lane et al. was based on 52 patients with chronic schizophrenia [12]. They reported a Cohen’s effect size (*d*) of 1.53. Our patients (with early psychosis) tend to have slightly lower mean PANSS total scores (65.0), with a standard deviation of 14.3 units and it is anticipated that the effect size will be smaller in this group. With an alpha value of 0.05, and power of 0.8, we wish to be able to confidently detect a difference in mean PANSS total score of at least 7 units. This will require 66 participants per group (n = 132). Over a 12-week period we predict an attrition rate of 15%. Thus, we will need to randomize approximately 160 subjects.

## Randomization and blinding procedure

Participants will be randomized to one of the treatment groups, using blocks of four via a computer-generated randomization table. Randomization will be stratified by five sites: (a) Metro North Hospital and Health Services (HHS), (b) Metro South HHS, (c) West Moreton HHS, (d) Gold Coast HHS and (e) Children’s Health Queensland HHS. Participants will be allocated to receive either active treatment or placebo in a 1:1 ratio.

The investigational products will be manufactured in accordance with current Good Manufacturing Practice (GMP) in a suitable Therapeutic Goods Administration (TGA) licensed facility. This same company (Pharmaceutical Packing Professionals) will hold the randomization code. Participants will be randomized strictly using a chronological process. Participants will be allocated a unique identification number, which will be linked to the specific site number. The randomization will be double-blind. An independent biostatistician will generate the randomization list, which will be provided to the manufacturer.

## Unblinding

The independent manufacturer will hold the closed randomization list and only the manufacturer will have the ability to unblind the allocation. In the case of an emergency when it is crucial the medical staff knows whether the participant is on sodium benzoate or placebo, participants will be provided with contact information (i.e. 24-hour telephone number) for unblinding the allocation. After study completion by all participants (last patient last visit), the participants will be notified as to which arm of the study they took part in.

## Drug administration

The “intervention” group will receive 1000 mg sodium benzoate (500 mg BD with meals-reminder aid) in the form of capsules and the “placebo group” will receive microcrystalline cellulose gelatine capsules with an identical appearance. Sodium benzoate will be dispensed to participants once consent has been obtained and after the screening phase and randomization. A delegated Research Pharmacist will dispense medication for all sites. The entire 12 weeks of study medication for each randomized participant will be provided to QCMHR delegated research staff. The study medication will then be distributed to the participant on a fortnightly basis by delegated research staff. There will be a total of seven dispensations per participant.

## Dose justification

The FAO/WHO Expert Committee on Food Additives established a preliminary acceptable daily intake of up to 5 mg/kg body weight for sodium benzoate as a food preservative [24]. This translates to 350 mg per day for a 70-kg person. With respect to individuals with urea cycle disorders, the recommended dose of sodium benzoate is between 250 and 500 mg/kg per day or approximately 17500 mg–35000 mg per day for a 70-kg person. It is noted that serious side effects at this dosage are rare and limited to occasional cases of anorexia and vomiting [31].

In the use of sodium benzoate for the treatment of schizophrenia, we are guided by the dose used by Lane and colleagues [12]. In the main study the investigators also described a pilot study (n = 7) dosing sodium benzoate between 250 and 1000 mg/d: in this study, 1000 mg/day resulted in the largest decrease in psychotic symptomatology, with no evident adverse reactions. In this patient group, a dose of 1000 mg per day was not associated with significantly more adverse events (AEs) compared to placebo. Treatment-emergent AEs included weight gain (n = 1), insomnia (n = 2), and tachycardia (n = 1). It was noted that these AEs were mild, brief and did not warrant medical treatment. The authors concluded these events were likely coincidental observations. Routine blood cell count, chemical analysis results and electrocardiogram after treatment remained unchanged and were all within normal ranges [12].

## Comparator justification

This study will use a placebo adjunct to routine care as a comparator condition. Routine care in this study is defined as “individualized combinations of psychopharmacology, behavioural interventions, rehabilitation and associated clinical services” in keeping with Queensland Health standards of care for psychosis. The Declaration of Helsinki affirms that placebo-controlled trials should only be used in the absence of existing proven therapy [32]. Therefore, the use of an adjunct therapy has been selected to ameliorate these ethical concerns as both the experimental and control groups will receive standard medical care (treatment as usual).

## Outcome measures

A battery of validated clinical measures, physical health measures (blood pressure, waist circumference, height, weight and body mass index (BMI)) and AEs will be collected at baseline and at weeks 2, 4, 6, 8, 10 and 12.

## Efficacy measures

The PANSS total score, a widely used scale for measuring symptom severity in patients with schizophrenia, will be used as the primary outcome measure.

Secondary outcome measures will include the following clinical assessments:

- The PANSS subscales, including Positive, Negative and General Psychopathology subscales
- The GAF, which is a numeric scale (1–100) used by mental health clinicians and physicians to subjectively rate the social, occupational, and psychological functioning of adults
- The CGI, which is used to measure symptom severity, treatment response and the efficacy of treatments in treatment studies of patients with schizophrenia
- The Hamilton Depression Rating Scale-17items (HDRS), which is a multiple-item questionnaire used to provide an indication of depression and a guide to evaluate recovery
- The AQOL, which is a 15-item instrument that measures five broad domains: psychological wellbeing, physical senses, social relationships, independent living and illness
- The OTI, which is a comprehensive evaluation tool that assesses participants’ drug and alcohol use over the previous month: drugs assessed include heroin, other opiates, alcohol, cannabis, amphetamines, cocaine, tranquillizers, barbiturates, hallucinogens, inhalants, tobacco and poly-drug use
- The APQ-6, which is a short 6-item measure designed to complement clinical assessment, to support dialogue between consumers and clinicians about recovery goals, and to allow monitoring of change over time at both individual and aggregated service levels [23]
- The IPAQ, which provides a common instrument that can be used to obtain internationally comparable data on health-related physical activity
- The SIMPAQ, which measures physical activity: it has been designed for use in various populations including clinical samples with high levels of sedentary behaviour
- The PAQ, which has been developed to assess attitudes: it specifically assesses general opinion on physical exercise, motivators and barriers to exercise
- The PGI-I, which is a self-report scale, whereby a patient rates their overall improvement as a result of a therapy

## Biomarkers

A 25-ml sample of whole blood will be collected via venipuncture at both baseline and final patient interviews. A portion of this sample will be used to assess the concentration of key analytes in the pathways related to sodium benzoate. D-amino acid oxidase is needed to catabolize D-alanine. D-alanine can be measured in serum with high-performance liquid chromatography (HPLC) [33]. Related species that could be altered by the intervention include L-alanine, D-serine and L-serine, glycine and glutamate. Collecting these measures will allow us to assess if sodium benzoate impacts on these analytes in the peripheral blood. The remainder of the blood sample will be stored indefinitely for future research. However, future studies involving the stored blood samples will require approval from a Human Research Ethics Committee (HREC). In the event that a person objects to any biological samples being collected and stored, they will not be excluded from participating in the trial.

## Frequency of visits and follow up

**Fig. 1:**
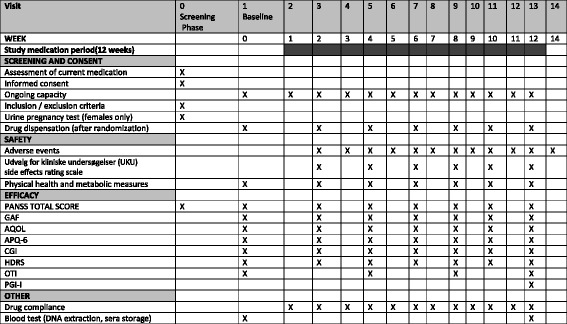
Schedule of visits and assessments. *GAF* Global Assessment of Functioning, *AQOL* Assessment of Quality of Life, *APQ* Activity and Participation Questionnaire, *CGI* Clinical Global Impression, *HDRS* Hamilton Depression Rating Scale, *OTI* Opiate Treatment Index, *PGI-I* Patients’ Global Impression of Improvement

Participants will be clinically assessed at baseline and weeks 2, 4, 6, 8, 10 and 12. The study team will also contact participants once a week in between one-to-one assessments by telephone (if no telephone then by face-to-face review). Detailed schedule of visits and assessments are presented in Fig. 1. This protocol was written following the SPIRIT checklist.

## Compliance monitoring and adherence

Participants will be requested to return all unused study medication (i.e. unopened blister packs or capsules not taken) and empty blister packs to the delegated research assistants. All unused supplies of study medication will be accounted for and documented by the designated Research Pharmacist. Compliance with study medication will be calculated at each visit by means of self-report and a capsule count. These data will be used to calculate compliance with medication for analysis purposes.

For adherence and compliance monitoring, participants will be contacted regularly by weekly telephone calls. To further aid compliance and as a quick visual guide, participants will be offered a diary to document the date and time of scheduled visits and tasks to be undertaken.

A current investigational product dispensing log will be kept and will contain the following information:

- the identification of the participant to whom the drug was dispensed
- the date(s) and quantity of the drug dispensed to the participant

The inventory will be available for inspection by study monitors during the study. Drug supplies, including participant returns, will be collected at the end of the study by the study monitors, while unused drug supplies may be destroyed by the Investigator or delegate, provided such disposition does not expose humans to risks from the drug. Records will be maintained by the Investigator of any such alternate disposition of the investigational product. These records will show the identification and quantity of each unit disposed of, the method of destruction (taking into account the requirements of local law), and the person who disposed of the investigational product. Where investigational product is destroyed on site, a record of destruction will be issued. Such records will be submitted to the Sponsor for reconciliation purposes.

## Case report form

A case report form (CRF) will be completed for each study participant, summarising all clinical screening and study data that are to be provided to the University of Queensland (Sponsor) for data analysis. In the CRF, participants will only be identified by their participant number in order to retain participant confidentiality. The completed CRFs will be retained by the Investigators for a period of at least 15 years or the maximum time frame as determined by local regulations, whichever is the longest.

## Study restrictions

There are no restrictions to participants during the study in terms of concomitant medications, exercise or ambulation. While sodium benzoate is often added to products rich in vitamin C such as orange juice, there has been concern that sodium benzoate may interact with high-dose vitamin C formulations to produce benzene (a known carcinogen) [34]. There is an absence of clear guidance on this matter, thus for the purposes of this protocol, we will adopt a conservative stance and require patients to avoid using vitamin C formulations that contain more than 500 mg/day (multivitamin tablets have lower doses of vitamin C and can be continued during the study, as can citrus fruits and food products that contain vitamin C naturally).

## Safety assessments

All patients recruited into this study will be current patients of Queensland Hospital and Health services. The study team will liaise with clinical staff to ensure that participants have undergone a routine physical health screen. Female participants will have a urinary pregnancy screen at baseline prior to inclusion, and during the study if indicated.

## Adverse events

The Investigator and designated study personnel will monitor each participant for AEs during the study. All AEs reported between consent and final follow-up visit will be recorded in the CRF. The investigator or designee will ask the participant non-leading questions in an effort to detect AEs e.g. “Have you felt unwell or different in any way since your last visit”. In addition, participants will be encouraged to spontaneously report any unusual feelings or sensations.

## Adverse events and serious adverse events

The investigator will be responsible for the detection and documentation of events meeting the criteria and definition of an AE or a serious adverse event (SAE). We have developed a separate database on AE reporting. We are using the Medical Dictionary for Regulatory Activities (MEDRA) coding system that includes System Organ Class (SOC), and following disease symptoms (lowest level term (LLT)) into the database for detailed record keeping of all related (and unrelated) AEs/SAEs. Each AE will be monitored until resolution or to the end of the 12-week protocol.

## Participant withdrawal by the Investigator

Patients will be withdrawn from the study by the Investigator, prior to completion of treatment, under the following conditions:

- Non-compliant with study medication for 7 consecutive days
- Development of an SAE assumed to be associated with the study medication
- Cessation of effective contraception or confirmed pregnancy
- Continual inability to provide informed consent

## Early termination and stopping rules

The study may be terminated prematurely by the Coordinating and or Principal Investigator or his/her designee and the Sponsor if: (a) the number and/or severity of AEs justify discontinuation of the study, (b) new data become available, which raise concern about the safety of the study drug, so that continuation might cause unacceptable risks to participants. Study closure advice will also be sent to the Reviewing Ethics Committee and relevant Governance Offices, and the TGA. The Australian Clinical Trial Registry entry will also be updated accordingly. Interim analysis may be performed if needed as aforementioned, and will be conducted by the University of Queensland (Sponsor) data analysis team.

## Participant reimbursement and compensation

Participants will be reimbursed for out-of-pocket expenses, inconvenience and time involved, by the provision of prepaid gift cards from a large retail outlet. We will provide a gift card worth Australian $40, at the end of week 2, mid-way through the protocol (week 6) and on completion (week 12) (total reimbursement Australian $120).

The clinical trial insurance will reimburse participants for costs of medical care that occur as a result of complications directly related to participation in this study. The Investigator and insurance company will be notified as soon as possible if this occurs or where a causal relationship cannot be excluded. All SAEs will be reported to the nominated insurance company. The University of Queensland (Sponsor) will enter into a Clinical Trial Agreement with each of the five Hospital and Health Services (HHSs) involved in the study, based on the standard Medicines Australia format

## Data management and monitoring

A screening log will be utilized to track potential participants and also record the counts of individuals approached, consented, meeting inclusion/exclusion criteria, withdrawn and completed (in keeping with standard requirements of the Consolidated Standards of Reporting Trials (CONSORT) diagram). The CRF will comprise the hard copy questionnaires, clinical assessments and measures. These de-identified data will be retained in a secure room, in a locked filing cabinet at each site. De-identified data from the CRFs will be entered into REDCap, which is a secure web-based application (encrypted to health-service standard, housed on a server behind the University of Queensland firewall) for building and managing online surveys and databases. Delegated research assistants will be trained in, and be responsible for, entering data into the database. Upon completion and resolution of monitoring and data management queries, the clinical trial database will be closed. All data will be exported into SAS software to enable statistical analysis. A copy of the database will be stored in a secure room in a locked filing cabinet separate from the CRFs.

The study coordinator or designated delegate will monitor data entered at each site and be responsible for resolving data entry errors and discrepancies. Data quality will be ensured by performing data entry checks for consistency between the CRF and the data entry into REDCap database. These checks will be performed during data entry so that discrepancies can be resolved immediately. A data manager will later perform additional checks for completeness and plausibility of data. Resultant queries will be raised and resolved electronically by the data manager and the study centre.

## Statistical analysis

All data will be analysed using SAS 9.4. The data will be provided to the biostatistician(s) employed by the Queensland Centre for Mental Health Research for analysis. We will identify demographic and clinical differences between the groups at baseline (chi-square test, Fisher exact test for nominal variables and Mann-Whitney test or independent sample *t* test for continuous variables). Efficacy will be assessed according to standard intention to treat (ITT) analytic procedures (i.e. for those who do not complete the 12-week study period, we will carry forward their last observation on the study outcomes). Mean changes in clinical assessment will be assessed using mixed-model repeated-measure (MMRM) methods with treatment, week and treatment-week interaction as fixed effects and intercept as the only random effect; the baseline value will be the covariant. The MMRM analyses will be performed using the SAS PROC mixed procedure. *P* values will be based on two-tailed tests with significance levels of 0.05.

## Monitoring and quality assurance

An independent Study Monitor will conduct study documentation review to monitor key features of the study prior to commencement and during and after study completion. These site visits will enable the Monitor to maintain current, personal knowledge of the study through review of the CRFs, to compare CRF entries against the electronic database (REDCap) and to discuss the conduct of the study with the Investigator. The Monitor will be responsible for monitoring adherence to the approved study protocol, regulatory compliance including GCP and completion of the CRF.

## Data Safety Monitoring Board

An independent Data Safety Monitoring Board (DSMB) will be established specifically to monitor safety data and study trends throughout the duration of the trial to determine if continuation of the trial is appropriate scientifically and ethically.

## Protocol amendments

Any amendments to the protocol will be submitted to the appointed HREC by the Chief Investigator for approval. Any approved amendments by the appointed HREC will be forwarded by the Chief Investigator for submission to each Research Governance Office. If a protocol amendment requires changes to the informed consent form, the revised informed consent form, prepared by the Chief Investigator, will be approved by the reviewing ethics committee and site governance officers. While the amendment affects the ongoing suitability of the study at a participating site, the Research Governance Office will determine the ongoing suitability based on the amendment submitted. The present version is 1.2, 31 October 2015.

## Discussion

The Cadence BZ study is the first clinical trial established as part of the wider Cadence Clinical Trials platform [35]. This project, funded by the National Health and Medical Research Council (NHMRC), aims to build capacity in clinician trials. To this end, a multidisciplinary working party was established in 2014, to guide the direction of future clinical research. Treatments that were safe and acceptable to consumers and caregivers were prioritized - thus interventions were chosen that involve compounds such as food preservatives (e.g. sodium benzoate) and that involved augmentation of standard treatments. A website has been developed to provide information to participants and their families, and for clinicians (www.cadencetrials.com).

Sodium benzoate appears a promising compound from several perspectives. It impacts on neurotransmitter pathways of interest to schizophrenia. For example, recent large genome-wide association studies have identified common variants in genes related to the N-methyl-D-aspartate (NMDA) neurotransmitter system (https://www.ncbi.nlm.nih.gov/pmc/articles/PMC4112379/ and https://www.ncbi.nlm.nih.gov/pmc/articles/PMC4361495/). The preliminary data from clinical trials looks promising. It involves a compound that has an attractive safety profile, and one that is acceptable to participants. We have based the dose of sodium benzoate (1000 mg/day) on the most recent clinical trial [12]. However, it is feasible that alternative doses may be optimal. Our study will not be able to explore this issue.

## Trial status

The study is currently recruiting participants. Recruitment commenced on 14 August 2015. Currently 50 participants have been randomized.

## Additional files

Additional file 1: Table S1. SPIRIT 2013 checklist. (DOC 126 kb)

Additional file 2: Table S2. Model consent from (adult). (DOCX 139 kb)

## Abbreviations

AE: Adverse event
APQ: Activity and Participation Questionnaire
AQOL: Assessment of Quality of Life
BD: Twice daily
BMI: Body mass index
CGI: Clinical Global Impression
CIB: Clinical Investigators’ Brochure
CRF: Case report form
CTN: Clinical trial notification
DAAO: D-amino acid oxidase
ES: Effect size
FAO: Food and Agriculture Organization of the United Nations
FDA: Food and Drug Administration
GAF: Global Assessment of Functioning
GCP: Good Clinical Practice
HDRS: Hamilton Depression Rating Scale
HHS: Hospital and Health Service
HREC: Human Research Ethics Committee
IEC: Independent Ethics Committee
IPCS: International Programme on Chemical Safety
NHMRC: National Health and Medical Research Council
NMDA: N-methyl-D-aspartate
NOAEL: No observable adverse effect level
OTI: Opiate Treatment Index
PANSS: Positive and Negative Syndrome Scale
PGI-I: Patients’ Global Impression of Improvement
PK: Pharmacokinetic
SAE: Serious adverse event
SANS: Scale for the Assessment of Negative Symptoms
SD: Standard deviation
SPIRIT: Standard Protocol Items: Recommendations for Interventional Trials
TAU: Treatment as usual
TGA: Therapeutic Goods Administration
WHO: World Health Organization
