# Supplementary material for: The efficacy of sodium benzoate as an adjunctive treatment in early psychosis - CADENCE-BZ: study protocol for a randomized controlled trial
Source: Trials. 2017 Apr 7;18:165. doi: 10.1186/s13063-017-1908-5 (PMC5383965; doi:10.1186/s13063-017-1908-5)
Supplement: Supplementary file 2 — Model consent from (adult). (DOCX 139 kb) [file 13063_2017_1908_MOESM2_ESM.docx]

**Supplementary material S2: Model Consent Form (condensed)**


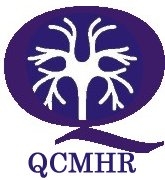

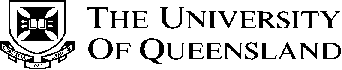
**
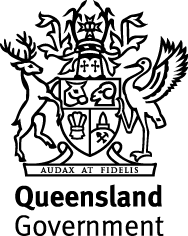
**

**INFORMATION SHEET**

**Title** The Efficacy of Sodium Benzoate as an Adjunctive Treatment in Early Psychosis (CADENCE-BZ)

**Sponsor** The University of Queensland

## **Coordinating Principal Investigator** Professor John McGrath

**Principal Investigator** A/Professor James Scott

**Location**

**Protocol** CADENCE-BZ

*This Participant Information and Consent Form is 13 pages long. Please make sure that you have all of the pages.*

Introduction

You are invited to take part in a multisite clinical trial (CADENCE-BZ). This is because you are being treated for a mental illness in an Early Psychosis clinic. The clinical trial is testing a new add-on treatment for those with early psychosis. Before you decide if you wish to consent to your participation we would like you to understand why the study is being done, what it will involve and how your information will be used. Please take time to read the following information carefully and if appropriate discuss it with friends, family and your doctor. One of our team will go through the information sheet with you and answer any questions you have. Please ask questions about anything that you do not understand or want to know more about.

Participation in this research is voluntary. If you don’t wish to take part, you don’t have to. It is desirable that your doctor be advised of your decision to participate in this study. If you have a doctor we strongly recommend that you inform them of your participation in this study. Once you understand what the project is about and if you agree to take part in it, you will be asked to sign the Consent Form. By signing the Consent Form, you indicate that you understand the information and that you give your consent to participate in the clinical trial. You will be given a copy of the Participant Information and Consent Form to keep as a record……………………………………..

……………………………………………………………………………………………………………………………..

**Participant Consent Form**

**Study Title** The Efficacy of Sodium Benzoate as an adjunctive treatment in Early Psychosis (CADENCE-BZ)

- I have read (or had read to me), the Information Sheet and I understand the purpose of the clinical trial, what is involved, what data is being collected, any possible risks, inconveniences or discomforts involved, and what will be done with the data upon completion of the clinical trial.
- I have been given the time and opportunity to ask questions about the clinical trial and any

questions I have asked have been answered clearly and to my satisfaction. I have also been given the opportunity to discuss this clinical trial with a person not connected to the clinical trial.

- I understand that all information provided by me is treated as strictly confidential and will only be shared with the clinical trial team and not be released by the clinical trial team unless required to do so by law.
- I agree that research data gathered for the clinical trial can be published as long as my name, or any identifying data, will not be used in any publication.
- I know that I may withdraw from the trial at any time without having to give any reason or affecting my current or future medical treatment.
- I understand I will receive a copy of the participant information and signed consent form to keep.
- I understand and consent to those regulatory authorities and other organisations referred to in the participant information having access to my confidential information.
- I agree to participate in this research and give my consent voluntarily.

**In addition: (initial next to your response)**

- I give permission for a member of the clinical trial team to recontact me within the next 5 years regarding possible participation in further mental health research. Yes No
- I give permission for a member of the clinical trial team to notify the participant’s primary care physician (provided that such a physician can be identified for the participant) and treating Psychiatrist of their participation in the study. Yes No
- I give consent for my blood to be taken at the time points specified for the purpose of this clinical trial. Yes No
- I give consent for my blood samples to be stored and used for unspecified future testing.

Yes No

- I give consent for the research team to review my health outcomes via my medical records (paper and electronic) and Health Research Databases during the course of the current study. Yes No

______________________________________________ ________________

Printed Name of Participant Initial

______________________________________________ ________________

Signature of Participant Date (participant to date)

_______________________________________________ _________________

Signature of Witness Date (witness to date)


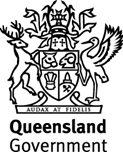

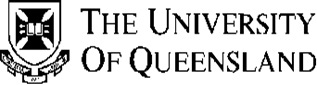

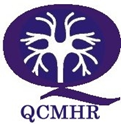


**REVOCATION OF CONSENT FORM**

**Full Project Title**: The Efficacy of Sodium Benzoate as an Add-On Treatment in Early Psychosis (CADENCE-BZ)

I hereby wish to WITHDRAW my consent to participate in the clinical trial described above and understand that such withdrawal WILL NOT affect my current or future treatment.

As part of my original consent I agreed that my data would be used as part of the study, regardless of my decision to withdraw. However I have provided a blood sample and request that my sample be (initial all that apply):

- used for the purposes of this research project and/or;
- stored for future use as outlined in the participant information sheet
- destroyed after use for this research project
- destroyed immediately and not used for this research project or for future research projects

Participant’s Name (printed) ……………………………………………………. Initial ………………..

Signature…………………………… Date……………………

Researcher’s Name (printed)…………………………………………………….

Signature…………………………… Date……………………
